# Decreases in procalcitonin and C-reactive protein are strong predictors of survival in ventilator-associated pneumonia

**DOI:** 10.1186/cc5036

**Published:** 2006-09-06

**Authors:** Renato Seligman, Michael Meisner, Thiago C Lisboa, Felipe T Hertz, Tania B Filippin, Jandyra MG Fachel, Paulo JZ Teixeira

**Affiliations:** 1 Department of Internal Medicine, Universidade Federal do Rio Grande do Sul, Rua Ramiro Barcelos 2400 – 4o Andar, 90035-003, Porto Alegre, Brasil; 2 Hospital de Clínicas de Porto Alegre, Rua Ramiro Barcelos, 2350, 90035-003, Porto Alegre, Brasil; 3 Hospital of Dresden-Neustadt, Industriestrasse 40, D-01129 Dresden, Germany; 4 Department of Statistics, Universidade Federal do Rio Grande do Sul, Av. Bento Gonçalves, 9500 – Prédio 43-111, 91509-900 Porto Alegre, Brasil; 5 Universidade Federal do Rio Grande do Sul, Centro Universitário Feevale, Rodovia RS 239, 2755, 93352-000. Novo Hamburgo, Brasil

## Abstract

**Introduction:**

This study sought to assess the prognostic value of the kinetics of procalcitonin (PCT), C-reactive protein (CRP) and clinical scores (clinical pulmonary infection score (CPIS), Sequential Organ Failure Assessment (SOFA)) in the outcome of ventilator-associated pneumonia (VAP) at an early time point, when adequacy of antimicrobial treatment is evaluated.

**Methods:**

This prospective observational cohort study was conducted in a teaching hospital. The subjects were 75 patients consecutively admitted to the intensive care unit from October 2003 to August 2005 who developed VAP. Patients were followed for 28 days after the diagnosis, when they were considered survivors. Patients who died before the 28th day were non-survivors. There were no interventions.

**Results:**

PCT, CRP and SOFA score were determined on day 0 and day 4. Variables included in the univariable logistic regression model for survival were age, Acute Physiology and Chronic Health Evaluation (APACHE) II score, decreasing ΔSOFA, decreasing ΔPCT and decreasing ΔCRP. Survival was directly related to decreasing ΔPCT with odds ratio (OR) = 5.67 (95% confidence interval 1.78 to 18.03), decreasing ΔCRP with OR = 3.78 (1.24 to 11.50), decreasing ΔSOFA with OR = 3.08 (1.02 to 9.26) and APACHE II score with OR = 0.92 (0.86 to 0.99). In a multivariable logistic regression model for survival, only decreasing ΔPCT with OR = 4.43 (1.08 to 18.18) and decreasing ΔCRP with OR = 7.40 (1.58 to 34.73) remained significant. Decreasing ΔCPIS was not related to survival (*p *= 0.59). There was a trend to correlate adequacy to survival. Fifty percent of the 20 patients treated with inadequate antibiotics and 65.5% of the 55 patients on adequate antibiotics survived (*p *= 0.29).

**Conclusion:**

Measurement of PCT and CRP at onset and on the fourth day of treatment can predict survival of VAP patients. A decrease in either one of these marker values predicts survival.

## Introduction

Ventilator-associated pneumonia (VAP) is defined as pneumonia occurring more than 48 hours after endotracheal intubation and the initiation of mechanical ventilation. When a clinical diagnosis of VAP is suggested by a new or progressive pulmonary infiltrate associated with fever, an increased white blood cell count and purulent tracheobronchial secretion [1,2], efforts directed towards the achievement of a microbial diagnosis of VAP by invasive or non-invasive techniques are justified [3].

The mortality rate for VAP ranges from 24% to 50% and can reach 76% in some specific settings or when lung infection is caused by high-risk pathogens [4]. A body of evidence shows that inadequate antimicrobial treatment is an important determinant of mortality [5-8]. Adequacy of antimicrobial therapy is usually assessed on the third day of treatment, on the basis of clinical parameters and microbiological identification.

Markers of the inflammatory response and their kinetics have been studied in the prediction of outcomes in sepsis [9,10] and VAP [11]. In particular, procalcitonin (PCT) has been evaluated as a marker of sepsis and infection. Severe generalized bacterial infections with systemic manifestations are associated with increased serum levels of PCT. In contrast, viral infections, localized bacterial infections, or inflammatory reactions of non-infectious origin do not, or only moderately, increase PCT levels [12,13]. Some studies describe PCT as a predictor of severity in sepsis, antimicrobial efficiency and hospital mortality [14-17]. Differential diagnosis and antibiotic treatment as well can be improved by using this marker [18,19].

Considering the variability of PCT levels, it is possible to theorize that increasing levels, more than a high livel at onset, may indicate persistent infection activity, whereas decreasing values suggest resolution. We undertook a study to assess the prognostic value of the kinetics of PCT, C-reactive protein (CRP), and the clinical scores clinical pulmonary infection score (CPIS) [20], Sequential Organ Failure Assessment (SOFA) [21], and Acute Physiology and Chronic Health Evaluation (APACHE) II [22], in the outcome of VAP at an early time point, when adequacy of antimicrobial treatment is evaluated.

## Materials and methods

The study was conducted in the clinical/surgical 26-bed intensive care unit (ICU) of the Hospital de Clínicas de Porto Alegre (HCPA), a tertiary-care–teaching institution with 744 hospital beds.

All patients consecutively admitted to the ICU suspected of VAP were eligible for this prospective observational cohort study. Patients at least 18 years old were recruited. Exclusion criteria were a previous diagnosis of AIDS or neutropenia below 500 cells/ml. Pneumonia was considered ventilator-associated when it occurred after 48 hours of mechanical ventilation and was judged to not have been incubating before starting mechanical ventilation. VAP was considered early-onset when it occurred during the first four days of mechanical ventilation and late-onset when it developed five or more days after the initiation of mechanical ventilation [23]. APACHE II was calculated during the first 24 hours of admission to ICU. Patients were considered immunosuppressed when they had received chemotherapy within the preceding 45 days, or had neutropenia of less than 1,000/mm^3^.

Diagnosis of pneumonia was suspected when a patient developed a new and persistent radiographic infiltrate plus two of the following: (1) body temperature more than 38°C or less than 36°C; (2) white blood cells more than 11,000 or less than 4,000/mm^3 ^(3) macroscopically purulent tracheal aspirate [24]. Purulent endotracheal aspirate was defined on inspection by the assistant team. The axillary temperature used was the highest in the previous 24 hours before the inclusion on the study.

Chest X-ray, arterial blood gases, complete blood count, creatinine, total bilirubin, albumin, PCT and CRP were obtained by the that time VAP was suspected (D0) and repeated on the fourth day of treatment (D4). Quantitative endotracheal aspirate (QEA) was obtained on D0, repeated on the third day after the diagnosis (D3) and then weekly. Sterile endotracheal aspirates were obtained with a suction catheter adapted to a mucus collector without saline instillation, and two samples of hemocultures were collected from different veins with a 15-minute interval before starting antimicrobial treatment.

CPIS [25], modified as described by Singh and colleagues [20], was calculated on the basis of data on D0 and D3. Patients were assumed to have VAP when the CPIS was 7 points or more. CPIS was calculated with data from D0, adding points for microbiological results and progression of pulmonary infiltrate on a new chest X-ray on D3. To calculate CPIS on D3, data from D3 were used to study the kinetics of the modified CPIS.

For a diagnosis of VAP, there should be no evidence of another medical condition to which the presenting symptoms, signs or radiological findings could be attributed. A SOFA score was calculated on D0 and D4. QEA was considered positive when values were at least 10^5 ^colony-forming units/ml.

All patients with a clinical suspicion of VAP, later confirmed by a CPIS of at least 7 and fulfilling inclusion criteria, were included and received empirical antimicrobial therapy on D0. The choice of antibiotics and changes rested solely with the critical care team or primary service caring for the patient. Modifications to empirical therapy were based on the results of QEA and hemocultures. Mechanical ventilation, physiotherapy and airway management were performed in accordance with a standard protocol in all patients.

Patients' progress was followed until the 28th day (D28) after the diagnosis of VAP, when they were considered survivors. Patients who died before D28 were non-survivors. Patients discharged from the ICU before D28 were also considered survivors. All patients with VAP were reviewed by one of the investigators to confirm the diagnosis on the basis of predetermined criteria.

Seventy-five patients were enrolled from October 2003 to August 2005 and constituted the study population. The research protocol was reviewed and approved by the Human Research Committee from the HCPA, and informed written consent was obtained from patients' representatives before enrollment. The study protocol conforms to the ethical guidelines of the Declaration of Helsinki.

Trained investigators collected data on D0, D3, D4, and weekly until D28. Recorded data included age, sex, cause of ICU admission, arterial partial pressure of oxygen/fraction of inspired oxygen (PaO_2_/FiO_2_), APACHE II score, SOFA score, CPIS, co-morbidities including chronic obstructive pulmonary disease, whether active smoker, history of congestive heart failure, history of malignancy, immunosuppression, albumin, use of histamine type-2 receptor (H2) antagonist, use of proton pump inhibitor, use of corticosteroids, dialysis, central vein catheterization, urinary tract catheterization, duration of mechanical ventilation, duration of stay in ICU before VAP, cardiopulmonary resuscitation, intubation (orotracheal versus nasotracheal), and tracheotomy.

Adequacy of the empirical antimicrobial treatment was recorded on the basis of microbiological results. Adequate antibiotic therapy was defined as coverage of all the pathogens isolated (from QEA culture or from blood), by at least one antimicrobial administered by the onset of VAP, determined by the sensitivity pattern in the antibiogram [26]. Treatment was considered adequate when cultures were negative.

PCT was determined with the commercially available immunoluminometric assay (Brahms PCT LIA; Brahms Diagnostika, Berlin, Germany) with an analytical sensitivity of 0.1 ng/ml and analyzed with a Lumat LB 9507 Luminometer (Berthold, Bad Wildbad, Germany). Blood was drawn when a diagnosis of VAP was clinically suspected, before empirical antibiotic treatment was started. Samples of serum were prepared and frozen immediately after blood was drawn, then stored at -80°C in the HCPA research laboratory. Assays were performed in batches at the end of the study period.

CRP was measured by nephelometry (Bade Behring, Marburg, Germany), routinely determined at the HCPA laboratory.

### Kinetics definitions

Dichotomized Δ was calculated by the formula Δ = D4 - D0. Therefore ΔPCT = PCT_D4 _- PCT_D0_, ΔCRP = CRP_D4 _- CRP_D0_, and ΔSOFA = SOFA_D4 _- SOFA_D0_.

CPIS was calculated on D0 and D3. Consequently, ΔCPIS = CPIS_D3 _- CPIS_D0_.

Δ > 0 means increasing values and Δ ≤ 0 means decreasing values.

### Microbiological processing

Endotracheal samples were initially analyzed with Gram stain. They were rejected if there were more than ten squamous epithelial cells per low-power field (magnification × 100), requiring a new sample [27]. Samples considered acceptable were mixed in a 1:1 proportion with *N*-acetylcysteine, mechanically liquefied and homogenized with a vortex mixer for two minutes. After incubation for two hours at room temperature, samples were again vortex-mixed for 30 s and serially diluted in sterile 0.9% saline solution to obtain final concentrations of 1:100 and 1:10,000. Aliquots of 0.1 ml were plated on chocolate agar. Depending on the Gram stain results, samples also were plated on sheep blood agar, azide blood agar or MacConkey agar. All plates were incubated at 35°C overnight in a 5% carbon dioxide incubator, except for those in MacConkey agar, which were incubated in normal atmosphere without carbon dioxide. Isolates were assessed within 24 and 48 hours and were characterized by colony morphology and Gram stain. Microorganisms were identified by standardized laboratory methods. For plates inoculated with 1:100 dilution, the presence of 5 colonies was considered to show 10^4^, 50 colonies 10^5^, and 500 colonies 10^6 ^colony-forming units/ml. In plates inoculated with 1:10,000 dilution, the presence of 5 colonies was considered to show 10^6 ^colony-forming units/ml.

### Statistical analysis

Continuous baseline data are expressed as means ± SD. Categorical variables were compared with the χ^2 ^test. Continuous kinetics data are expressed as medians (range). ΔPCT, ΔCRP and ΔSOFA were categorized as increasing or unchanged/decreasing. The Kruskal–Wallis test was used to compare groups for continuous variables. For these analyses, two-tailed tests and *p *≤ 0.05 were considered statistically significant. Logistic regression analysis was used to determine the relation of risk factors to clinical outcome. In the multivariable model we considered significant variables with biological importance. Variables with *p *< 0.20 in univariable logistic regression were entered into the multivariable model. In the multivariable model we considered as significant those variables with *p *< 0.05. SPSS 11.0 for Windows (SPSS Inc., Chicago, IL, USA) was used for statistical analysis.

## Results

Baseline characteristics at the inclusion of the 75 VAP patients, stratified as survivors or non-survivors, are given in Table 1. Microbiological identification in QEAs is shown in Table 2.

**Figure 1:**
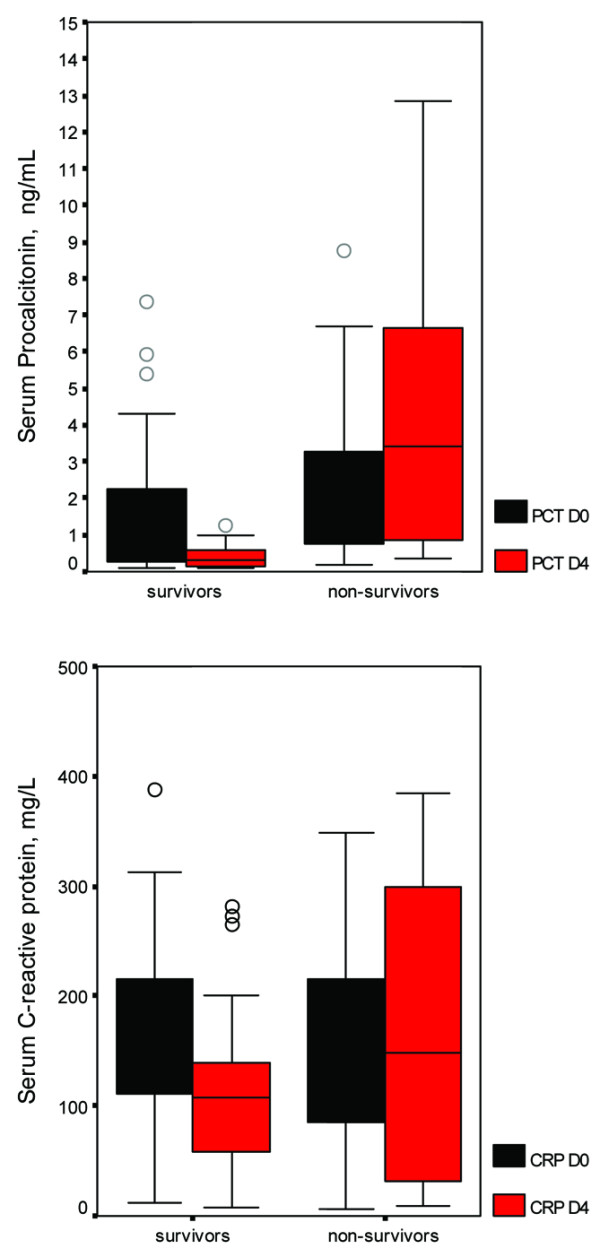
Kinetics of serum PCT (top) and CRP (bottom) in survivors and non-survivors. Box plots represent the 25th and 75th centiles; the internal horizontal line shows the median and whiskers show the 10th and 90th centiles. Circles represent outliers. Comparing between groups, *p *= 0.003 in D0 and *p *< 0.001 in D4 for procalcitonin (PCT); *p *= 0.77 in D0 and *p *= 0.14 in D4 for C-reactive protein (CRP).

Kinetic data on PCT and CRP from D0 to D4 are shown in Figure 1. PCT levels were lower in survivors on D0 (*p *= 0.003) and on D4 (*p *= 0.001). PCT levels increased in non-survivors but not in survivors (Table 3). Decreasing ΔPCT was not related to adequacy of antibiotic treatment on the basis of QEA results on D0 (*p *= 0.76). CRP levels showed no difference between survivors and non-survivors on D0 (*p *= 0.77) and on D4 (*p *= 0.14). Decreasing ΔCRP was not related to adequacy of antibiotic treatment based on QEA results on D0 (*p *= 0.13). CPIS did not discriminate survivors from non-survivors on D0 (*p *= 0.32) or on D3 (*p *= 0.45). Decreasing ΔCPIS was not related to survival (*p *= 0.59); neither was CPIS < 6 on D3 (*p *= 0.79). Decreasing ΔCPIS and CPIS < 6 on D3 were not related to adequacy of antibiotic treatment (*p *= 1.00 and *p *= 0.55, respectively). Patients who died before D4 could not have kinetics determined and are classed as missing cases. Fifteen patients were not included in the kinetics analysis. Eight patients died and one patient left the ICU before D4. Data on six patients were missing as a consequence of a logistic flaw. The evolution of clinical scores and laboratory parameters are presented in Table 3.

Variables included in the univariable logistic regression model for survival were age, APACHE II, decreasing ΔSOFA, decreasing ΔPCT, and decreasing ΔCRP. The results are shown in Table 4. Survival was directly related to decreasing ΔPCT with odds ratio (OR) = 5.67 (95% confidence interval 1.78 to 18.03), *p *= 0.003; decreasing ΔCRP with OR = 3.78 (1.24 to 11.50), *p *= 0.02; decreasing ΔSOFA with OR = 3.08 (1.02 to 9.26), *p *= 0.05; and APACHE II score with OR = 0.92 (0.86 to 0.99), *p *= 0.02. Age was not significant, but *p *= 0.10 was a reason for inclusion in the multivariable model.

The multivariable logistic regression model for survival included the variables from the univariable analysis. Only decreasing ΔPCT with OR = 4.43 (1.08 to 18.18), *p *= 0.04, and decreasing ΔCRP with OR = 7.40 (1.58 to 34.73), *p *= 0.01, remained significant (Table 4).

Adequacy of antimicrobial treatment based on microbiological data of QEA on D0 did not discriminate survivors from non-survivors (*p *= 0.29; Table 5).

The influence of septic status on the kinetics of PCT, CRP and SOFA is shown in Table 6. Increasing ΔPCT was more frequent than sepsis and severe sepsis in septic shock, and decreasing ΔPCT occurred more frequently in sepsis and severe sepsis; however, these results were not statistically significant (*p *= 0.12). There was no difference in the performance of ΔCRP (*p *= 0.96) and ΔSOFA (*p *= 0.97) in all three statuses.

None of the patients included in the study achieved immunosuppression criteria. Only 17.3% of patients received corticosteroids, without statistical significance between survivors and non-survivors (*p *= 0.23) and corticosteroid dosage between groups (*p *= 0.25).

## Discussion

Our results showed that, on D0 and D4, PCT levels and SOFA score differentiated survivors from non-survivors. Decreasing values of CRP and decreasing values of PCT were able to predict, respectively, a sevenfold and fourfold greater chance for patients with VAP to survive.

CRP is used as a parameter to support the diagnosis of infection [9]. Yentis and colleagues [10] demonstrated that a decrease in CRP by 25% or more from the previous day's level was a good indicator of resolution of sepsis, with a sensitivity of 97%, a specificity of 95% and a predictive value of 97%. The decrease in CRP preceded clinical resolution of sepsis and was more likely to occur in patients with less severe sepsis than in those with severe sepsis or septic shock [10]. In our results, absolute CRP levels could not differentiate survivors from non-survivors on D0 and D4 (*p *= 0.77 and 0.14, respectively). Nevertheless, similarly to the results of Yentis and colleagues in patients with sepsis, in our VAP patients the decrease of CRP levels was significantly predictive of survival, with OR = 7.40.

We assessed the correlation of CPIS changes from D0 to D3 with survival. The kinetics of CPIS from D0 to D3 was chosen to compare with published data. Luna and colleagues [26] studied 63 patients with clinical evidence of VAP and bacteriologic confirmation by bronchoalveolar lavage or blood cultures. In their sample, CPIS fell progressively in the population as a whole, and the decrease in CPIS was significant in survivors but not in non-survivors. When CPIS was less than 6 at 3 or 5 days after VAP onset, mortality was lower than in the remaining patients. They considered these differences to be related to the finding that patients receiving adequate therapy had a slight fall in CPIS, whereas those receiving inadequate therapy did not. However, their mortality rate was not statistically significant: 69.2% for patients treated with inadequate antibiotic treatment and 46.0% for patients on adequate antibiotic treatment (*p *= 0.238). Their mortality rate in all patients was 50.8%. Those results are in partial contrast with ours. In our sample, serial measurements of modified CPIS on D0 and D3 could not differentiate between survivors and non-survivors (*p *= 0.44 for D0; *p *= 0.43 for D3). We could not correlate decreasing ΔCPIS with survival (*p *= 0.79); CPIS < 6 on D3 was also not correlated with survival (*p *= 0.59).

We assessed the possible correlation of changes in CPIS from D0 to D3 with adequacy of antibiotic treatment on the basis of microbiological results of QEA on D0. We could not correlate decreasing ΔCPIS with adequacy of antibiotic treatment (*p *= 1.00) and CPIS < 6 on D3 with adequacy of treatment (*p *= 0.55). Neither decreasing ΔPCT and ΔCRP was related to adequacy of antibiotic treatment on the basis of QEA results on D0 in our sample. We also could not correlate survival with adequacy of antibiotic treatment: 50.0% of the 20 patients treated with inadequate antibiotics survived, and 65.5% of the 55 patients on adequate antibiotics survived (*p *= 0.29). There was a trend to correlate adequacy with survival, but the lack of statistical significance may represent a type 2 error.

Multiple organ dysfunction syndrome is associated with mortality. Vincent and colleagues [21] demonstrated that multiple organ dysfunction and high SOFA scores for any individual organ were associated with increased mortality. In their sample, the SOFA score increased in 44% of the non-survivors but in only 20% of the survivors (*p *< 0.001) in patients who stayed at least 1 week in the ICU. Our results with SOFA score were similar to those: the SOFA score was higher in non-survivors on D0 (*p *= 0.002) and D4 (*p *= 0.002; Table 3) and decreasing SOFA scores were predictive of survival, with OR = 3.08 (*p *= 0.05) in the univariable, but not in the multivariable, logistic regression (*p *= 0.54).

In our results, PCT levels were significantly higher in non-survivors on D0 (*p *= 0.003) and D4 (*p *= <0.001). Furthermore, the decrease in PCT levels was significantly predictive of survival, with OR = 4.43. Other studies on patients with VAP have reported higher PCT levels in non-survivors than in survivors [28,29]. In a study with children with severe bacterial infection, Assicot and colleagues [12] reported that serum PCT values decreased rapidly during antibiotic therapy.

We analyzed the influence of septic status on the kinetics of PCT, CRP and SOFA. Increasing ΔPCT was more frequent in patients with septic shock than in septic and severely septic patients. There was a trend of more frequent decreasing ΔPCT sepsis and severe sepsis, but these results were not statistically significant (*p *= 0.12) and may be attributed to sample size. There was no difference in the performance of ΔCRP and ΔSOFA in all three statuses (*p *= 0.96 and *p *= 0.97, respectively) in our sample.

Although high PCT levels at the onset of sepsis have been reported to be associated with lethal outcome [30], this observation was not corroborated by Meisner and colleagues [14]. They compared PCT and CRP at different SOFA scores during the course of sepsis and multiple organ dysfunction syndrome. Measurement of PCT during multiple organ dysfunction syndrome provided more information about the severity and the course of disease than that of CRP. Higher SOFA scores were associated with significantly higher plasma PCT concentrations, whereas CRP was elevated irrespective of the scores observed. In a similar manner to our results, they found an increase in PCT levels after day 4 in non-survivors (*p *< 0.01). In that study, a rapid decline of PCT levels in patients who recovered and survived was also observed, whereas CRP increased for several days even after recovery and discharge of the patient from the ICU.

Changes in biological markers levels may indicate a modification in clinical status. In a recent study, Luyt and colleagues [11] assessed the value of PCT kinetics as a prognostic marker during VAP in 63 patients, with measures on days 1, 3 and 7. Unfavorable outcomes were death, recurrence of VAP, or occurrence of extrapulmonary infection requiring antibiotic treatment during the first 28 days of VAP. PCT levels in the sample generally decreased from D1 to D7 but increased in patients with unfavorable outcome. For PCT analysis, the study by Luyt and colleagues used the time-resolved amplified cryptate emission technology, an expensive apparatus not available worldwide. We analyzed PCT with a luminometry assay, which is less expensive and commercially available. This strategy has the inconvenience of being less sensitive than the research technology, but it may offer a cost-effective option. Our study design also had some distinctions: our main outcome was survival and we assessed change in PCT over four days, which may be clinically more relevant because this is the time frame during which VAP treatment is frequently reassessed [31].

Considering the mortality rate of VAP, it is highly desirable to have early laboratory markers to predict survival or the necessity to reassess initial empirical antimicrobial therapy. In our sample, decreasing ΔSOFA, ΔPCT and ΔCRP were significantly predictive of survival in univariable analysis, but the multivariable regression model maintained only ΔPCT and ΔCRP as independent predictors for survival, as early as in D4. Patients may express different serum levels of markers when exposed to bacterial toxins, and this stimulation may be multifactorial. The advantage of kinetics was that, independently of an absolute value, decreasing values were related to survival; this outcome is suggestive of a decrease in stimulus to inflammation and a decrease in exposure to bacterial toxins.

## Conclusion

Measurement of PCT and CRP at onset and the fourth day of treatment can predict the survival of patients with VAP. A decrease in either of these marker values predicts survival. The identification of those with good outcome as early as on day four could possibly help to ensure the adequacy of antimicrobial therapy. Further studies with a larger sample are necessary to establish whether a combination of marker kinetics can be used to guide antimicrobial therapy, especially in cases in which microorganisms are not identified.

## Key messages

• Survival is directly related to decreasing levels of procalcitonin and C-reactive protein in ventilator-associated pneumonia.

## Abbreviations

APACHE = Acute Physiology and Chronic Health Evaluation; CPIS = clinical pulmonary infection score; CRP = C-reactive protein; ICU = intensive care unit; HCPA = Hospital de Clínicas de Porto Alegre; OR = odds ratio; PCT = procalcitonin; QEA = quantitative endotracheal aspirate; SOFA = Sequential Organ Failure Assessment; VAP = ventilator-associated pneumonia.

## Competing interests

MM has received remuneration for holding lectures on the topic of inflammation markers by BRAHMS-AG, Germany. The authors declare that there are no further competing interests.

## Authors' contributions

RS developed the study design and coordinated its implementation. RS, MM and PJZT participated in interpretation/discussion of results and drafted and revised the manuscript. RS, TCL and FTH were responsible for patient recruitment as well as data collection. RS and TBF carried out laboratory tests. RS, PJZT and JMGF carried out the statistical analysis. All authors read and approved the final manuscript.

## Figures and Tables

**Table 1:** Baseline characteristics of 75 patients who developed ventilator-associated pneumonia

Parameter	Survivors (*n *= 46)	Non-survivors (*n *= 29)	Total (*n *= 75)	*p*
Age (years)^a^	58 ± 14)	64 ± 16	61 ± 15	0.09
APACHE II score^a^	18 ± 6	22 ± 9	19 ± 7	0.02
Albumin level (mg/dl)^a^	2.8 ± 0.6	2.5 ± 0.5	2.7 ± 0.6	0.02
Male sex (percentage)	67.4	44.8	58.7	0.06
Origin (percentage)				0.23
Medical	52.2	69.0	58.7	
Surgical	47.8	31.0	41.3	
Onset (percentage)^b^				0.59
Early onset	21.7	27.6	24.0	
Late onset	78.3	72.4	76.0	
COPD (percentage)	17.4	27.6	21.3	0.39
Congestive heart failure (percentage)	17.4	27.6	21.3	0.39
Malignancy (percentage)	13.1	13.8	13.3	1.00
H2 antagonist (percentage)	67.4	55.2	62.7	0.33
Proton pump inhibitor (percentage)	21.7	37.9	28.0	0.19
Corticosteroids (percentage)	13.1	24.1	17.3	0.23
Dialysis (percentage)	10.9	17.3	13.3	0.50
Smoker (percentage)	37.8	44.8	40.5	0.63
Intubation (percentage)				0.39
Orotracheal	84.8	89.7	87.8	
Nasotracheal	2.2	3.5	2.7	
Tracheotomy	13.1	3.5	9.5	
Septic status (percentage)				0.04
Sepsis	67.4	13.8	46.7	
Severe sepsis	28.3	34.5	30.7	
Septic shock	4.4	51.7	22.7	

APACHE, Acute Physiology and Chronic Health Evaluation; COPD, chronic obstructive pulmonary disease. ^a^Results are means ± SD. ^b^Early onset is defined as occurring during the first four days of mechanical ventilation, and late onset as occurring five or more days after mechanical ventilation.

**Table 2:** Microbiological identification and survival in quantitative endotracheal aspirate in 75 patients with ventilator-associated pneumonia

Microorganisms	Survivors (*n *= 58)	Non-survivors (*n *= 34)	Total (*n *= 92)^a^
*Pseudomonas aeruginosa*	9 (15.5)	6 (17.7)	15 (16.3)
Oxacillin-resistant *Staphylococcus aureus*	8 (13.8)	6 (17.7)	14 (15.2)
Oxacillin-sensitive *Staphylococcus aureus*	7 (12.1)	1 (3.0)	8 (8.7)
*Stenotrophomonas maltophilia*	3 (5.2)	3 (8.8)	6 (6.5)
*Acinetobacter *sp.	4 (6.9)	1 (3.0)	5 (5.4)
*Enterobacter *sp.	4 (6.9)	1 (3.0)	5 (5.4)
*Klebsiella pneumoniae*	2 (3.5)	3 (8.8)	5 (5.4)
*Haemophilus *sp.	4 (6.9)	0	4 (4.4)
*Escherichia coli*	1 (1.7)	2 (5.9)	3 (3.3)
*Citrobacter koseri*	2 (3.5)	0	2 (2.2)
*Proteus mirabilis*	2 (3.5)	0	2 (2.2)
Other Gram-negative nonfermenting bacilli	1 (1.7)	0	1 (1.1)
*Acinetobacter junii*	1 (1.7)	0	1 (1.1)
*Streptococcus agalactiae*	1 (1.7)	0	1 (1.1)
*Serratia *sp.	1 (1.7)	0	1 (1.1)
*Moraxella *sp.	1 (1.7)	0	1 (1.1)
*Streptoccocus pneumoniae*	1 (1.7)	0	1 (1.1)
*Burkholderia cepacea*	0	1 (3.0)	1 (1.1)
Unidentified	6 (10.4)	10 (29.4)	16 (17.4)

^a ^In 17 patients more than one microorganism was identified.

Quantitative endotracheal aspirate was deemed positive when ≥10^5 ^colony-forming units/ml. Not all percentages add up to 100 because of rounding. Results are given as frequency (percentage).

**Table 3:** Evolution of clinical scores and laboratory parameters in patients with ventilator-associated pneumonia

Parameter	Survivors (*n *= 45)	Non-survivors (*n *= 23)	*p*
Procalcitonin D0	0.58 (0.08–19.60)	2.18 (0.19–21.33)	0.003
Procalcitonin D4	0.30 (0.08–36.19)	3.44 (0.39–17.00)	<0.001
C-reactive protein D0	160.0 (11.6–388.0)	167.5 (5.6–349.0)	0.77
C-reactive protein D4	108.0 (6.8–282.0)	148.0 (9.0–384.0)	0.14
SOFA_D0_	5 (0–12)	7 (2–20)	0.002
SOFA_D4_	4 (0–13)	7 (0–18)	0.002
CPIS_D0_^a^	8 (5–13)	8 (7–12)	0.32
CPIS_D3_	6 (2–12)	7 (3–10)	0.45

Results are shown as median (range). D0, day of VAP diagnosis; D3, 3 days after VAP; D4, 4 days after VAP diagnosis; SOFA, Sequential Organ Failure Assessment; CPIS, modified clinical pulmonary infection score. ^a^CPIS was calculated as described by Singh and colleagues.

**Table 4:** Odds ratios for survival in 75 patients with ventilator-associated pneumonia

Parameter	Univariable		Multivariable	
	OR (95% CI)	*p*	OR (95% CI)	*p*
Age (years)	0.97 (0.84–1.01)	0.10	0.96 (0.91–1.01)	0.11
APACHE II score	0.92 (0.86–0.99)	0.02	0.91 (0.81–1.02)	0.09
ΔSOFA decreasing values	3.08 (1.02–9.26)	0.05	2.25 (0.48–10.46)	0.30
ΔCRP decreasing levels	3.78 (1.24–11.50)	0.02	7.40 (1.58–34.73)	0.01
ΔPCT decreasing levels	5.67 (1.78–18.03)	0.003	4.43 (1.08–18.18)	0.04

The results show a univariable and multivariable logistic regression analysis of parameter kinetics. APACHE, Acute Physiology and Chronic Health Evaluation; SOFA, Sequential Organ Failure Assessment; CRP, C-reactive protein; PCT, procalcitonin; OR, odds ratio; CI, confidence interval.

**Table 5:** Cross-tabulation of adequacy of treatment with survival in 75 patients with ventilator-associated pneumonia

Adequacy of treatment	Survivors (*n *= 46)	Non-survivors (*n *= 29)	Total (*n *= 75)	*p*
Adequate	36 (78.3)	19 (62.1)	55 (72)	0.29
Inadequate	10 (21.7)	10 (37.9)	20 (28)	

Results are numbers of patients; percentages are given in parentheses.

**Table 6:** Cross-tabulation of PCT, CRP and SOFA kinetics with septic status in patients with VAP

Kinetics	Sepsis	Severe sepsis	Septic shock	*p*
ΔPCT ≤ 0 (*n *= 42)	23 (74.2)	14 (70.0)	5 (41.7)	
ΔPCT > 0 (*n *= 21)	8 (25.8)	6 (30.0)	7 (58.3)	
Total (*n *= 63)	31	20	12	0.12
ΔCRP ≤ 0 (*n *= 45)	22 (71.0)	15 (68.2)	8 (66.7)	
ΔCRP > 0 (*n *= 20)	9 (29.0)	7 (31.8)	4 (33.3)	
Total (*n *= 65)	31	22	12	0.96
ΔSOFA ≤ 0 (*n *= 49)	24 (72.7)	16 (72.7)	9 (69.2)	
ΔSOFA > 0 (*n *= 19)	9 (27.3)	6 (27.3)	4 (30.8)	
Total (*n *= 68)	33	22	13	0.97

Results are numbers of patients; percentages are given in parentheses. VAP, ventilator-associated pneumonia; PCT, procalcitonin; CRP, C-reactive protein; SOFA, Sequential Organ Failure Assessment.

## References

[B1] Meduri GU (1995). Diagnosis and differential diagnosis of ventilator-associated pneumonia. Clin Chest Med.

[B2] Rello J, Paiva JA, Baraibar J, Barcenilla F, Bodi M, Castander D, Correa H, Diaz E, Garnacho J, Llorio M (2001). International Conference for the Development of Consensus on the Diagnosis and Treatment of Ventilator-associated Pneumonia. Chest.

[B3] Sanchez-Nieto JM, Torres A, Garcia-Cordoba F, El-Ebiary M, Carrillo A, Ruiz J, Nunes ML, Niederman M (1998). Impact of invasive and noninvasive quantitative culture sampling on outcome of ventilator-associated pneumonia: a pilot study. Am J Respir Crit Care Med.

[B4] Chastre J, Fagon JY (2002). Ventilator-associated pneumonia. Am J Respir Crit Care Med.

[B5] Dupont H, Mentec H, Sollet JP, Bleichner G (2001). Impact of appropriateness of initial antibiotic therapy on the outcome of ventilator-associated pneumonia. Intensive Care Med.

[B6] Kollef MH, Sherman G, Ward S, Fraser VJ (1999). Inadequate antimicrobial treatment of infections: a risk factor for hospital mortality among critically ill patients. Chest.

[B7] Luna CM, Vujacich P, Niederman MS, Vay C, Gherardi C, Matera J, Jolly EC (1997). Impact of BAL data on the therapy and outcome of ventilator-associated pneumonia. Chest.

[B8] Rello J, Gallego M, Mariscal D, Sonora R, Valles J (1997). The value of routine microbial investigation in ventilator-associated pneumonia. Am J Respir Crit Care Med.

[B9] Ugarte H, Silva E, Mercan D, De MA, Vincent JL (1999). Procalcitonin used as a marker of infection in the intensive care unit. Crit Care Med.

[B10] Yentis SM, Soni N, Sheldon J (1995). C-reactive protein as an indicator of resolution of sepsis in the intensive care unit. Intensive Care Med.

[B11] Luyt CE, Guerin V, Combes A, Trouillet JL, Ayed SB, Bernard M, Gibert C, Chastre J (2005). Procalcitonin kinetics as a prognostic marker of ventilator-associated pneumonia. Am J Respir Crit Care Med.

[B12] Assicot M, Gendrel D, Carsin H, Raymond J, Guilbaud J, Bohuon C (1993). High serum procalcitonin concentrations in patients with sepsis and infection. Lancet.

[B13] Nylen ES, Snider RH, Thompson KA, Rohatgi P, Becker KL (1996). Pneumonitis-associated hyperprocalcitoninemia. Am J Med Sci.

[B14] Meisner M, Tschaikowsky K, Palmaers T, Schmidt J (1999). Comparison of procalcitonin (PCT) and C-reactive protein (CRP) plasma concentrations at different SOFA scores during the course of sepsis and MODS. Crit Care.

[B15] Muller B, Becker KL, Schachinger H, Rickenbacher PR, Huber PR, Zimmerli W, Ritz R (2000). Calcitonin precursors are reliable markers of sepsis in a medical intensive care unit. Crit Care Med.

[B16] Pettila V, Hynninen M, Takkunen O, Kuusela P, Valtonen M (2002). Predictive value of procalcitonin and interleukin 6 in critically ill patients with suspected sepsis. Intensive Care Med.

[B17] Wanner GA, Keel M, Steckholzer U, Beier W, Stocker R, Ertel W (2000). Relationship between procalcitonin plasma levels and severity of injury, sepsis, organ failure, and mortality in injured patients. Crit Care Med.

[B18] Christ-Crain M, Jaccard-Stolz D, Bingisser R, Gencay MM, Huber PR, Tamm M, Muller B (2004). Effect of procalcitonin-guided treatment on antibiotic use and outcome in lower respiratory tract infections: cluster-randomised, single-blinded intervention trial. Lancet.

[B19] Harbarth S, Holeckova K, Froidevaux C, Pittet D, Ricou B, Grau GE, Vadas L, Pugin J (2001). Diagnostic value of procalcitonin, interleukin-6, and interleukin-8 in critically ill patients admitted with suspected sepsis. Am J Respir Crit Care Med.

[B20] Singh N, Rogers P, Atwood CW, Wagener MM, Yu VL (2000). Short-course empiric antibiotic therapy for patients with pulmonary infiltrates in the intensive care unit. A proposed solution for indiscriminate antibiotic prescription. Am J Respir Crit Care Med.

[B21] Vincent JL, de Mendonca A, Cantraine F, Moreno R, Takala J, Suter PM, Sprung CL, Colardyn F, Blecher S (1998). Use of the SOFA score to assess the incidence of organ dysfunction/failure in intensive care units: results of a multicenter, prospective study. Working group on 'sepsis-related problems' of the European Society of Intensive Care Medicine. Crit Care Med.

[B22] Knaus WA, Draper EA, Wagner DP, Zimmerman JE (1985). APACHE II: a severity of disease classification system. Crit Care Med.

[B23] Langer M, Cigada M, Mandelli M, Mosconi P, Tognoni G (1987). Early onset pneumonia: a multicenter study in intensive care units. Intensive Care Med.

[B24] Fabregas N, Ewig S, Torres A, El Ebiary M, Ramirez J, de la Bellacasa JP, Bauer T, Cabello H (1999). Clinical diagnosis of ventilator associated pneumonia revisited: comparative validation using immediate post-mortem lung biopsies. Thorax.

[B25] Pugin J, Auckenthaler R, Mili N, Janssens JP, Lew PD, Suter PM (1991). Diagnosis of ventilator-associated pneumonia by bacteriologic analysis of bronchoscopic and nonbronchoscopic 'blind' bronchoalveolar lavage fluid. Am Rev Respir Dis.

[B26] Luna CM, Blanzaco D, Niederman MS, Matarucco W, Baredes NC, Desmery P, Palizas F, Menga G, Rios F, Apezteguia C (2003). Resolution of ventilator-associated pneumonia: prospective evaluation of the clinical pulmonary infection score as an early clinical predictor of outcome. Crit Care Med.

[B27] Morris AJ, Tanner DC, Reller LB (1993). Rejection criteria for endotracheal aspirates from adults. J Clin Microbiol.

[B28] Brunkhorst FM, Al Nawas B, Krummenauer F, Forycki ZF, Shah PM (2002). Procalcitonin, C-reactive protein and APACHE II score for risk evaluation in patients with severe pneumonia. Clin Microbiol Infect.

[B29] Duflo F, Debon R, Monneret G, Bienvenu J, Chassard D, Allaouchiche B (2002). Alveolar and serum procalcitonin: diagnostic and prognostic value in ventilator-associated pneumonia. Anesthesiology.

[B30] Oberhoffer M, Vogelsang H, Russwurm S, Hartung T, Reinhart K (1999). Outcome prediction by traditional and new markers of inflammation in patients with sepsis. Clin Chem Lab Med.

[B31] (2005). Guidelines for the management of adults with hospital-acquired, ventilator-associated, and healthcare-associated pneumonia. Am J Respir Crit Care Med.

